# Clinical features of children with nontyphoidal Salmonella bacteremia: A single institution survey in rural Japan

**DOI:** 10.1371/journal.pone.0176990

**Published:** 2017-06-09

**Authors:** Yoshihiro Aoki, Katsuhiko Kitazawa, Hironobu Kobayashi, Masayoshi Senda, Yukie Arahata, Riu Homma, Yudai Watanabe, Akihito Honda

**Affiliations:** 1 Department of Pediatrics, Asahi General Hospital, Chiba, Japan; 2 Department of Clinical Laboratory, Asahi General Hospital, Chiba, Japan; University of Liverpool, UNITED KINGDOM

## Abstract

Nontyphoidal *Salmonella* (NTS) can cause bacterial enterocolitis. Although some children with NTS infection develop bacteremia, its clinical manifestations have not been discussed adequately. Therefore, we examined children with NTS bacteremia. We retrospectively examined the medical records of 15 patients aged less than 15 years. *Salmonella* spp. were detected in the blood cultures of these patients between 1991 and 2014. We divided an additional sample group of 34 patients diagnosed with an NTS infection between 2005 and 2014, into 2 groups. Group bacteremia (B) included patients in whose blood cultures *Salmonella* spp. were detected, and group non-bacteremia (NB) included patients in whom *Salmonella* infection was not detected. We compared each group using Wilcoxon test and Fisher’s exact test. The number of patients with fever, diarrhea, or abdominal pain was 15 (100%), 13 (87%), and 9 (60%), respectively, in the first sample of patients. However, vomiting and bloody stool were observed in only 5 patients (33%). More than 70% of patients exhibited a reduced white blood cell count, while C-reactive protein levels were variable in the patients. *Salmonella* spp. were detected via stool culture in 10 patients (67%). Diarrhea persisted for more than 4 days more frequently in group B than group NB (p = 0.004). The number of patients whose fever persisted for more than 4 days was significantly higher in group B than group NB (p = 0.030). Therefore, if NTS bacteremia is suspected, blood cultures should be collected and antibiotics should be initiated in cases with diarrhea or fever for more than 4 days. Furthermore, a negative stool culture result does not preclude the possibility of NTS bacteremia.

## Introduction

Although the burden of nontyphoidal *Salmonella* (NTS) infection differs between developing and developed countries, NTS is still an important cause of bacterial enterocolitis worldwide [1]. *Salmonella* ser. Typhimurium is reported to be the first, and *Salmonella* ser. Enteritidis the second most common NTS serovar [2]; however, this can vary, and serovars may be unique to different ecological and geographic regions. For example, in sub-Saharan Africa, a novel *Salmonella* Typhimurium multilocus sequence type, ST313, has been described, which currently accounts for the invasive diseases [3]. In Japan, *Salmonella* ser. Infantis is the most frequently detected serotype of *Salmonella* in seafood, followed by the serotypes Typhimurium, Schwarzengrund, and Manhattan [4]. In addition to NTS infections in the form of food-borne infections following the consumption of chicken eggs, there has been an increase in NTS infections from pets and other animals, especially reptiles [5]. NTS infections mainly produce gastrointestinal symptoms, which are usually self-limiting. However, approximately 9% of patients are said to develop bacteremia [6]. While considering the indication of antibiotics against bacterial enterocolitis, it is important to understand the clinical features of NTS bacteremia. Nevertheless, there are only a few detailed studies on the clinical manifestations of the infection owing to the low frequency of its occurrence. In this study, we retrospectively examined the clinical manifestations of NTS bacteremia in children, at a Japanese regional core hospital and discussed the indications for blood culture tests and antibiotics following the onset of the infection.

## Materials and methods

We retrospectively examined the medical records of 16 pediatric patients who had *Salmonella* spp. detected in their blood cultures between 1991 and 2014 (24 years) at a Japanese regional hospital. One patient with *Salmonella* ser. Typhi was excluded; hence, 15 patients were included in the study. We assessed the demographic and clinical characteristics of the patients, such as age, sex, predicted infection pathway, clinical symptoms (e.g., fever, diarrhea, abdominal pain, vomiting, and bloody stools), laboratory findings, bacteriological characteristics, treatment, complications, and outcomes.

Venous blood was cultured by direct inoculation into broth (BACTEC Peds Plus, Japan BD) and subsequently, automated growth detection instruments (BD BACTEC 9120 Blood Culture System, BD BACTEC 9240 Blood Culture System, and BD BACTEC FX Blood Culture System) were employed in accordance with standard methods [7]. We used Trypticase Soy Agar with 5% Sheep Blood (TSA II) (Japan BD), Bromothymol Blue (BTB) lactose agar (Japan BD), and chocolate agar (Kyokuto Pharmaceutical Industrial Co, Ltd.) to culture the microorganisms. Salmonella Shigella agar (Japan BD) was used for stool cultures. Bacterial identification and sensitivity testing was done using BD Phoenix (Japan BD). Anti-*Salmonella* serum (DENKA SEIKEN, Tokyo, Japan) was used for typing. Ours is not an institution for specialized microbiology testing; thus, the bacterial laboratory in our hospital could not identify the causative serotype of salmonella.

In addition, we examined 50 patients under 15 years of age who were diagnosed with an NTS infection by stool or blood culture in the same facility, between 2005 and 2014 (10 years). Sixteen patients were excluded because we did not collect their blood cultures. The remaining 34 patients were divided into two groups: group bacteremia (B), which included patients who had *Salmonella* spp. detected in their blood cultures, and group non-bacteremia (NB), which included patients who did not have *Salmonella* spp. detected in their blood cultures. To compare the clinical manifestations of the infection between the two groups, we used JMP 9 statistical software (SAS Institute Inc., Cary, NC, USA) as well as Wilcoxon test and Fisher’s exact test.

We obtained the consent and authorization of the Clinical Research Ethics Committee of Asahi General Hospital to perform this study. Patient information was anonymized and de-identified prior to analysis, so that patient consent was not necessary.

## Results

### (i) Patient characteristics

A summary of the demographic and clinical characteristics of the 15 children with NTS bacteremia is shown in Table 1. The annual number of cases was zero to three, and there was no change in the number of cases compared with the previous 12 years before 2002 and the following 12 years after 2002. Eight patients were male, and seven patients were female. The median age (n = 15) was 5 years (interquartile range [IQR] = 1–9 years), and included three patients aged less than 1 year (20%), seven patients between 1 and 6 years of age (47%), three patients between 7 and 12 years of age (20%), and two patients more than 13 years of age (13%). Although most patients were children younger than 6 years, some patients older than 6 years were also observed. There were no patients with underlying immune deficiency disorders or malnutrition.

**Table 1:** A summary of the demographic and clinical characteristics of the 15 children with NTS bacteremia.

No.	Year	Age (years)	Age (months)	Sex	Diagnosis	Sero group	Stool culture	Predicted route of infection	History of antibiotic therapy	Duration of diarrhea (days)	Highest number of diarrhea (/day)	Duration of fever (days)	Highest temperature (°C)	Abdominal pain	Vomiting	Bloody stool	WBC (/μL)	CRP (mg/dL)	AUS	Initial antibiotics	Duration of antibiotic therapy (days)	Hospitalization (days)
1	1991	5	65	F	Bacteremia, Enteritis	O9, H-G	Positive	Unknown	-	3	>10	3	39.3	+	+	-	6,800	12	NA	ABPC+CTRX	17	19
2	1994	1	15	F	Bacteremia, Enteritis	O7, H-r	Positive	Unknown	-	4	>10	3	38.5	-	+	+	10,100	2.1	Normal	FOM+CTX	11	9
3	1999	9	119	M	Bacteremia, Enteritis	O4, H-eh, en	Negative	Unknown	+	4	>10	5	40.7	+	+	-	8,200	15.3	LA, BWT	FOM	33	26
4	1999	7	86	F	Bacteremia	O7, H-g	Negative	Unknown	-	0	0	10	39.0	-	-	-	4,700	2.1	Normal	CTX	15	10
5	2000	0	0.5	F	Bacteremia, Enteritis	O4, H-i, 1, 2	Positive	Mother (carrier)	-	1	>10	5	38.0	-	-	-	19,000	1.1	NA	ABPC+CTX	10	14
6	2000	0	2	M	Bacteremia, Enteritis	O9, H-g	Positive	Unknown	-	1	>10	2	39.7	-	-	-	6,500	4.3	Normal	ABPC+CTX	14	17
7	2000	0	4	F	Bacteremia, Enteritis	O9, H-g	Positive	Unknown	-	30	>10	1	38.3	-	-	+	22,110	0.7	Normal	CTM	14	16
8	2003	6	72	M	Bacteremia, Enteritis	O9, H-g	Positive	Unknown	-	6	8	6	40.1	+	+	-	9,300	10.4	LA	CTX	7	11
9	2005	11	135	M	Bacteremia, Enteritis	O7, H-G	Positive	Unknown	-	10	7	8	38.6	-	-	-	5,800	5.5	Normal	FOM	14	10
10	2008	14	168	M	Bacteremia, Enteritis	O7, H-g	Negative	Raw egg	+	18	>10	8	39.6	+	-	+	4,300	2.1	LA	FOM	14	9
11	2010	2	32	M	Bacteremia, Enteritis	O7, H-r	Negative	Unknown	-	6	6	6	39.7	+	-	+	7,600	7	LA, BWT	FOM	14	0
12	2010	5	64	M	Bacteremia, Enteritis	O9, H-g	Negative	Unknown	-	14	>10	5	39.0	+	-	-	6,000	0.8	LA, BWT	CTRX	14	4
13	2010	2	30	F	Bacteremia, Enteritis	O4, H-b	Positive	Raw egg	-	1	1	2	39.1	+	-	-	34,300	0.6	Normal	FOM	14	4
14	2011	1	19	M	Bacteremia, Enteritis	O4, H-1	Positive	Turtle	+	6	6	7	39.1	+	-	+	9,400	1.9	Normal	AMPC	14	9
15	2014	15	183	F	Bacteremia, Enteritis	O4, H-e, n	Positive	Unknown	+	0	0	6	38.0	+	+	-	8,600	5.2	LA	CFPN-PI	14	9

WBC, white blood cell; CRP, C-reactive protein; AUS, abdominal ultrasonography; NA, not available; LA, lymph adenopathy; BWT, bowel wall thickening; ABPC, ampicillin; CTRX, ceftriaxone; FOM, fosfomycin; CTX, cefotaxime; CTM, cefotiam; AMPC, amoxicillin; CFPN-PI, cefcapene pivoxil

Regarding the predicted route of infection, two patients (13%) were probably infected via ingestion of chicken eggs, one patient (6.7%) via contact with a red-eared slider turtle, and one patient (6.7%) via contact with a carrier. It was either difficult or impossible for us to identify the route of infection in more than two thirds of the cases. Antibacterial drugs had been administered to four patients (27%) prior to the collection of blood or stool cultures.

### (ii) Clinical manifestations

The number of patients with fever, diarrhea, or abdominal pain was 15 (100%), 13 (87%), and nine (60%), respectively. However, vomiting and bloody stools were observed in only five patients (33%). Although more than 70% of patients had a white blood cell (WBC) count <10 000 cells/μL, the WBC count of one patient was >30 000/μL. The levels of C-reactive protein (CRP) were variable among patients; however, in the three patients with a high WBC count, the CRP levels were low. Abdominal ultrasonography (AUS) was performed in 13 of 15 patients; abdominal lymph adenopathy (LA) and bowel wall thickening (BWT) were observed in about 20–40% of patients. *Salmonella* spp. were detected via stool culture in 10 patients (67%). Histories of prior antibiotic administration were obtained for two of five patients in whom stool cultures showed negative results. Third generation cephalosporins were selected for use as the first antimicrobial agent in more than 60% of the patients. In these 15 patients, complications of shock, meningitis, and osteomyelitis were not observed. All patients experienced improvement after treatment with antimicrobial agents.

### (iii) Drug resistance

The 15 strains of *Salmonella* spp. isolated exhibited high rates of resistance (approximately 40–100%) to the first and second-generation cephalosporins and aminoglycosides, while no resistant strains were observed during the use of the third generation cephalosporins and quinolones.

### (iv) Comparisons of group bacteremia (B) and group non-bacteremia (NB)

The results of the comparisons of group B and group NB are shown in Table 2 and S1 Table. The median age of group B (n = 7) was 5 years (IQR = 2–14 years). Five patients (71%) were male. The median age of group NB (n = 27) was 3 years (IQR = 2–7 years), and 20 patients (74%) were male. Diarrhea was observed in six patients (86%) from group B and 25 patients (93%) from group NB. The duration of diarrhea between onset and culture collection for patients in group B was longer than that of patients in group NB (6 days vs. 3 days, p = 0.060). Therefore, the number of patients whose diarrhea persisted for more than 4 days in group B was also significantly higher than that in group NB (Odds Ratio [OR] = 20.0, 95% confidence interval [CI] = 2.62–153, 71% vs. 11%, p = 0.004). Fever was observed in all patients in both groups. The duration of fever between onset and culture collection for group B was significantly longer than that for group NB (6 days vs 3 days, p = 0.002). Therefore, the number of patients whose fever persisted for more than 4 days in group B was also significantly higher than that in group NB (OR = 11.3, 95% CI = 1.08–109, 86% vs. 33%, p = 0.030). The rate of abdominal pain in group B was higher than that in group NB (OR = 4.80, 95% CI = 0.51–45.5, 86% vs. 56%, p = 0.21). Conversely, vomiting occurred less frequently in group B than in group NB (OR = 0.21, 95% CI = 0.02–1.97 14% vs. 44%, p = 0.21). In group NB, 1 patient (3.7%) developed a febrile seizure. There was no significant difference in WBC count and CRP levels between both groups. Serum lactate dehydrogenase (LDH) levels in group B were higher than that in group NB (436 IU/L vs 298 IU/L, p = 0.008). Hospitalization rates were not significantly different between both groups, and antimicrobial therapy was administered to 15 patients (56%), even in group NB. The rates of each O-antigen, O4, O7, O8, and O9, were 43%, 43%, 0%, and 14%, respectively, in group B. Whereas, the rates of these O-antigens were 37%, 19%, 7.5%, and 37%, respectively, in group NB. No significant difference in O-antigen rates was found between the two groups. Further identification tests on the bacteria were not performed.

**Table 2:** Comparisons between groups B and NB.

	Group B, bacteremia (n = 7)		Group NB, non-bacteremia (n = 27)		OR	95%CI	P value
Median age of years (IQR)	5	(2.0–14)	3	(2.0–7.0)	NA	NA	0.36
Male, *n* (%)	5	(71)	20	(74)	1.14	0.179–7.28	1.0
History of antibiotic therapy, *n* (%)	3	(43)	8	(30)	1.78	0.322–9.85	0.66
Diarrhea, *n* (%)	6	(86)	25	(93)	0.48	0.0370–6.21	0.51
Median days until examination (IQR)	6.0 (1.0–14)		3.0 (2.0–3.0)		NA	NA	0.06
≧4 days, *n* (%)	5	(71)	3	(11)	20	2.62–153	0.004
≧10 times/day, *n* (%)	2	(29)	16	(59)	0.225	0.0360–1.41	0.2
Fever, *n* (%)	7	(100)	27	(100)	NA	NA	NA
Median days until examination (IQR)	6.0 (5.0–8.0)		3.0 (2.0–4.0)		NA	NA	0.002
≧4 days, *n* (%)	6	(86)	9	(33)	11.3	1.18–109	0.030
≧39.5°C, *n* (%)	2	(29)	6	(26)	1.13	0.171–7.47	1.0
Abdominal pain, *n* (%)	6	(86)	15	(56)	4.8	0.506–45.5	0.21
Vomiting, *n* (%)	1	(14)	12	(44)	0.208	0.021–1.97	0.21
Bloody stool, *n* (%)	3	(43)	9	(33)	1.5	0.275–8.19	0.68
Complications, *n* (%)	0	(0)	1	(3.7)	NA	NA	1.0
Immunodeficiency, *n* (%)	0	(0)	0	(0)	NA	NA	1.0
WBC (/μl) (IQR)	7,600 (5,800–9,400)		9,400 (7,000–13,700)		NA	NA	0.31
WBC ≧10,000/μl, *n* (%)	1	(14)	12	(44)	0.208	0.021–1.98	0.21
CRP (mg/dl) (IQR)	2.1 (0.81–5.5)		2.7 (2.0–8.4)		NA	NA	0.21
CRP ≧5 mg/dl, *n* (%)	3	(43)	12	(44)	0.938	0.175–5.02	1.0
LDH (IU/L) (IQR)	436 (314–559)		298 (258–351)		NA	NA	0.008
AUS:BWT / LA, *n* (%)	4	(57)	12	(44)	1.67	0.311–8.93	0.68
Positive of stool culture, *n* (%)	4	(57)	27	(100)	NA	NA	0.006
Hospitalization, *n* (%)	6	(86)	22	(81)	1.36	0.132–14.0	1.0
Antibiotic therapy, *n* (%)	7	(100)	15	(56)	NA	NA	0.036

OR, Odds Ratio; CI, confidence interval; IQR, interquartile range; NA, not available; WBC, white blood cell; CRP, C-reactive protein; LDH, lactate dehydrogenase; AUS, abdominal ultrasonography; BWT, bowel wall thickening; LA, lymphadenopathy

## Discussion

In this study of 15 patients, NTS bacteremias were detected in older children of school age who were otherwise healthy. Although the number of patients with invasive nontyphoidal *Salmonella* infections has been significantly decreasing over the past 20 years in Japan [8], cases of infection still exist. Although the symptoms include diarrhea, fever, and abdominal pain in most patients, 14% of patients in this study have diarrhea. Vomiting and bloody stools were observed in one-third of patients. As some patients did not test positive for the bacteria in stool cultures and many patients did not exhibit an increase in the WBC count, these were not considered important indicators of NTS bacteremia.

It has been suggested that infancy, immune deficiency, malnutrition, malaria, and anemia are risk factors for NTS bacteremia. Furthermore, it is recommended that blood cultures be performed to detect NTS infections in infants [9]. In this study, however, the patients were children of varying ages, who did not have any significant underlying diseases, such as immune deficiency. Consequently, we should be careful while considering NTS bacteremia in pediatric patients, even in school-age children, regardless of the underlying diseases. Although the sources of NTS infection includes foods, such as chicken eggs; contaminated water; contact with animals, such as reptiles, rodents, dogs, and cats; and hospital-acquired infections [10], in this study, the predicted infection routes in most cases were unknown. Therefore, even if the source of the infection in suspected NTS cases cannot be identified, the possibility of NTS infection must not be precluded as a differential diagnosis. Reptile-related salmonellosis, as noted recently, has been reported to mainly affect infants and has a high rate of hospitalization and invasive infection [5].

In previous cases, pediatric NTS bacteremia was shown to result from gastroenteritis in 75% of patients. These patients had a good prognosis [11]. In addition, patients with NTS bacteremia, when compared with patients not diagnosed with bacteremia, reported a longer symptomatic period, a higher erythrocyte count, and a higher serum LDH level, while also exhibiting reduced dehydration, symptom severity, and vomiting [12]. In this study, patients in group B showed a significantly longer duration of fever and diarrhea than those in group NB. In addition, serum LDH levels of patients in group B were significantly higher than that of patients in group NB. However, it was difficult to arrive at a diagnosis of NTS bacteremia using only these parameters. Therefore, it is important to consider the possibility that the symptoms are the result of bacteremia, despite a normal WBC count.

During NTS infections, bacteria adhere to and invade the intestinal epithelium and produce toxins, such as cholera enterotoxin, resulting in electrolyte and water flooding of the alimentary canal. The bacteria are ingested by M cells in Peyer's patches of the distal ileum and proximal colon, and then survive within macrophages of Peyer's patches, intestinal lymph nodes, and the parenteral reticuloendothelial system, which sometimes results in bacteremia [13, 14]. Both differences in the host's immune system and gene differences in each bacterial virulence factor are believed to be factors that increase the risk of bacteremia. Moreover, *S*. ser. Choleraesuis, *S*. ser. Heidelberg, and *S*. ser. Dublin can easily enter the bloodstream [15–17]. Clinically, *S*. ser. Heidelberg is believed to produce bacteremia more frequently [18]. Recently, a common core of virulence genes for invasive NTS has been identified [19], and there may be further unidentified virulence factors that increase invasiveness. Our results show that it is difficult to identify common characteristics and host factors influencing NTS bacteremia in children. Unfortunately, in this survey, a detailed identification of the bacteria was not performed. Similarly, in general clinical practice, it would be difficult to determine the names of the bacteria in cases of infection early on during the diagnosis. Although there may be no special value in solely identifying each O-antigen, we may suspect highly invasive *salmonella* spp. from the overall information, for example, *S*. ser. Heidelberg (O4), *S*. ser, Choleraesuis (O7), and *S*. ser. Dublin (O9). Furthermore, we may be able to exclude the cases of *Salmonella* ser. Typhi (O9) or *Salmonella* ser. Paratyhi A (O2), which have a different clinical course from non-typhoidal *Salmonella* by checking O-antigens.

The results of drug susceptibility tests indicated that bacterial infections were resistant to the first and second generation cephalosporins in 40–100% of cases and to aminoglycosides in 40–60% of cases. In Taiwan, ampicillin resistance was documented in 40% of cases [20]. NTS in the United States exhibits resistance against 4 or more antibiotics [21]. These patterns of resistance are similar to that observed in this study. Conversely, in India, more than half of the NTS were reported to have produced extended spectrum beta(β) lactamase (ESBL), a potent resistance mechanism that evades antimicrobial therapies [22]. This suggests the need to be conscious of the possibility of future drug resistance in this region. While a third generation cephalosporin is the standard antimicrobial therapy for treating sepsis and is appropriate considering drug susceptibility, it is necessary to select antibiotics on the basis of susceptibility in each regional area and to be careful of clinical test results, as reported in this study.

It is currently believed that it is necessary to perform a blood culture even if patients are afebrile or are in a good general condition, when *Salmonella* spp. grow in stool cultures in infants who are <3 months of age [9]. Furthermore, it is also recommended to start antibiotic treatment for an NTS infection in infants who are <3 months of age, who have chronic gastrointestinal diseases, malignant tumors, hemoglobinopathy, HIV infection, or immune deficiency, or who take immune suppressants [23]. Among 50 patients with NTS infections confirmed via stool cultures over the past 10 years in our facility, blood cultures were performed for 34 patients (68%). Although there were only seven patients (14%) with bacteremia among the 50 patients, there was no difference in the hospitalization rate between groups B and NB. Furthermore, antimicrobial drugs were used in more than half of the patients in group NB. This result suggests that there are many patients whose systemic condition is poor enough to lead to sepsis even in cases that are not complicated by bacteremia. In NTS infections, it may be clinically difficult to maintain the administration of antibiotics in patients without a confirmed risk.

It has been suggested that stool cultures should be performed for patients if they experience diarrhea for more than 5 days [9]. In this study, however, the median duration of diarrhea was 3 days in the NB group. Moreover, current standards indicate that a stool culture should be conducted if the patient presents with fever, abdominal pain, and a poor general condition. Although, in this study, stool cultures were negative for five of 15 patients with NTS bacteremia, only two out of five patients had taken antibiotics in advance. Although there may be technical issues with taking samples, it is possible for the number of intestinal bacteria to decrease below the sensitivity level when patients develop bacteremia. In instances such as these, it is of particular importance to perform a diagnosis via blood cultures.

This study has several limitations. First, we included children for whom we performed at least one blood culture; therefore, both patient groups were possibly more severely ill than patients with NTS enterocolitis in the community. Second, although some variables were significant in the bivariate analysis, we did not perform a multivariate analysis because of the small number of patients. In addition, this was a retrospective analysis and may not be applicable across all regions.

This research was conducted in a region of Japan, which is a developed country. NTS bacteremia should be recognized as a type of bacteremia that develops in healthy children of all ages. Therefore, clinicians must consider performing a blood culture even if patients do not exhibit leukocytosis, particularly among patients with a history of eating chicken eggs or having contact with reptiles, and who develop fever, diarrhea, and abdominal pain. In particular, clinicians must obtain blood cultures and initiate antibiotics if they strongly suspect NTS bacteremia in cases with diarrhea and fever that persist for 4 days or more. Furthermore, a negative stool culture result does not preclude the possibility of NTS bacteremia. Our results may be generalizable at least to other developed countries since Japan is a developed country.

## Supporting information

S1 Table Minimal data set for comparisons between groups B and NB. (XLSX)
